# The ability of *Clostridium bifermentans* strains to lactic acid biosynthesis in various environmental conditions

**DOI:** 10.1186/2193-1801-2-44

**Published:** 2013-02-11

**Authors:** Katarzyna Leja, Kamila Myszka, Katarzyna Czaczyk

**Affiliations:** Department of Biotechnology and Food Microbiology, Poznan University of Life Sciences, Wojska Polskiego 48, 60-627 Poznan, Poland

**Keywords:** Carbon source, Glycerol, Lactic acid, pH

## Abstract

*Clostridium bifermentans* strains, isolated from a manure, were examinated for their ability to produce lactic acid from PY medium with glycerol under different pH conditions and when PY medium was supplemented with saccharides such as fructose, sorbitol, glucose, mannose, mannitol, maltose, xylose, raffinose, and arabinose. In the last test performed, the ability of investigated strains to produce lactic acid from mixed carbon source (glycerol plus saccharide) was checked. The strains of *Cl. bifermentans*, designated as CB 371, CB 374, and CB 376 grew and produced lactic acid on PY medium irrespective of pH and the carbon source used. The optimal lactic acid production on PY medium with glycerol was obtained at pH of 7.0 in case of CB 371 and 376 (19.63 g/L and 16.65 g/L, accordingly) and at pH 8.0 in case of CB 374 (13.88 g/L). The best productivity of lactic acid on PY media by CB 371, CB 374, and CB 376 (above 30 g/L) was observed when mannitol was used as a carbon source. The mixed carbon source did not increase productivity of lactic acid by *Cl.* bifermentans. The yield of lactic acid was approximately equal to the yield of lactic acid obtained on the medium with only glycerol and lower than in medium with only mannitol. Thus, from the environmental point of view it is more beneficial to use the medium with waste-type material only, such as glycerol.

**Electronic supplementary material:**

The online version of this article (doi:10.1186/2193-1801-2-44) contains supplementary material, which is available to authorized users.

## Introduction

*Cl. bifermentans* was first isolated by Tissier and Martelly in 1902. A taxonomic relationship to *Cl. sordelli*, isolated first in 1922, resulted in the symptomatic fact that both strains were identified as one species (Brooks & Epps 1958). As late as in 1963, *Cl. bifermentans* and *Cl. sordelli* were distinguished as separate species of the genus *Clostridium*. As a main factor whose influence was taken into consideration here was pathogenicity: *Cl. sordelli* was described as a pathogenic variant of non-pathogenic *Cl. bifermentans*. Additionally, these two bacterial species can be distinguished one from another in the urease-production test. By 1955, the idea of separating *Cl. bifermentans* and *Cl. sordelli* gained acceptance of researchers. The original isolate of *Clostridium bifermentans* was named *Bacillus bifermentans sporogenes* (Clark and Hall 1937), and later re-named *B. bifermentans* (Bergey et al. 1923), in accordance with the principle of binominal nomenclature (Brooks & Epps 1958). The main sources of *Cl. bifermentans* occur in water, soil, sewage (Nachman et al. 1989), sludge, and animal faeces (Wang et al. 2003).

*Cl. bifermentans* is able to produce a wide range of metabolites such as acetic, butyric and formic acids (Wu & Yang 2003), ethanol, butanol, aceton (Khanal et al. 2004), carbon dioxide, hydrogen, and nitrogen (Levin et al. 2006). However, the metabolic pathway of *Cl. bifermentans* has not been investigated in detail so far.

The aim of this work thus was to investigate the possibility of lactic acid production by *Cl. bifermentans* when, as carbon source, glycerol or other saccharides are added to the cultivation medium as well as under low- and high-pH stress on glycerol medium.

## Materials and methods

### Source of strains

*Cl. bifermentans* strains (KM 371, KM 374 and KM 376) were isolated from samples that were collected from a manure in the *Wielkopolska* Region, Poland. Samples were collected in sterile plastic jars and stored in refrigerator until experimentations. Liquid samples were then inoculated to the modified PY medium according to Biebl and Spöer (2002). The isolating process is described in more detail in Myszka et al. (2012).

### Cultivation medium

The modified PY medium consisted of (g/L): BactoPeptone 10; yeast extract 10; CaCl_2_, MgSO_4_ × 7H_2_O 0.96; K_2_HPO_4_ 2; NaHCO_3_ 20; NaCl 4 was used. As a source of carbon in the PY medium, glycerol (50 g/L) or saccharides such as fructose, sorbitol, glucose, mannose, mannitol, maltose, xylose, raffinose, and arabinose (50 g/L) (Sigma-Aldrich) were added. The pH of the PY medium without regulation is of the value of 8.6 using 10% solution of NaOH and HCl (Sigma Aldrich).

### Batch fermentation

A preculture was carried out in a 500 ml flask containing 300 ml PY medium with glycerol at 37^°^C for 24 h. It was inoculated into a 5 L bioreactor (Sartorius Stedim, Germany) with 3 L PY medium (with glycerol or, respectively, saccharide). According to Myszka et al. (2012), a blanket of a high-purity grade gas mixture of 5% O_2_ and 95% CO_2_ was maintained through 24 h. Gas flow rate was at up to 1.0 L/min only. During the first 24 h of cultivation the level of 5% of oxygen was automatically maintained (the stirrer speed varied between 200 and 500 rpm). After 24 h of the duration of the process the stirrer speed was regulated to a constant value of 200 rpm. The fermentation was run at 30°C for 7 days.

### Fermentation at various pH conditions

The experiments were carried out in the PY medium with glycerol. The pH was adjusted to 3, 5, 6, 7, 8, 9, 10, and 13 with 10 M KOH and 10 M HCl solutions. As a control pH the value of 8.6 was used. The aim of this step was to estimate the ability of the cells to survive low and high values of pH, and compare the levels of lactic acid and other metabolites produced in these conditions.

### Fermentation of saccharides

The ability of strains to produce acids and other metabolites from saccharide (50 g/L) such as fructose, sorbitol, glucose, mannose, mannitol, maltose, xylose, raffinose, and arabinose was examined in PY medium without glycerol. The carbohydrate solutions were sterilized by filtration and added to the PY medium without glycerol. In further experiments, bacteria were cultivated in the PY medium with mixed carbon sources – glycerol constituted 80% and one of the saccharides 20% of a carbon source. In the control experiment, only glycerol (50 g/L) was used.

In this step, the influence of a carbon source on lactic acid production and other metabolites was evaluated.

The yields of lactic acid (Y_LA_) were calculated as g lactic acid per g substrate. The calculation for Y_LA_ is shown as Eq. 1:

1 $$Y_{\text{LA}}=\frac{\text{LA}\left(g\right)}{\text{substrate}\left(g\right)}$$

### Analytical procedures

After fermentation the cell free supernatants were collected. The products were delineated with a high liquid performance chromatography (HPLC) technique. The Hewlett Packard system consisted of an auto sampler and a pump, and a refractive index detector was used. The analysis was performed isocratically at flow rate 0.6 mL/min. at 65°C, on a column Aminex HPX- 87H300 × 7.8 (Bio-Rad,USA). 0.5 mNH2SO4 as a mobile phase was also used. The standards were applied to identify peaks in chromatograms, and peak areas were measured to determine the samples’ concentration (ChemStation, Agilent, USA).

## Results

### Influence of pH value on the level of lactic acid production

During our research on 1,3-propanediol (1,3-PD) production from glycerol (Myszka et al. 2012; Leja et al. 2011) the ability of lactic acid synthesis by new isolated *Cl. bifermentans* strains was observed. The metabolite profile of the investigated strains, KM 371, KM 374, and KM 376, in microaerophilic conditions in PY medium with glycerol (pH 8.66) is presented in Table 1. In our subsequent experiment, we checked the influence of the pH value on the level of lactic acid production. Cells of KM 371, KM 374 and KM 376 were cultivated in PY medium with glycerol in pH value set at 3.0, 5.0, 6.0, 7.0, 8.0, 9.0, 10.0, and 13.0 with no pH control during fermentation. The control cultivation was carried out in pH arranged at 8.66 – a typical value of PY medium resulted from a large amount of NaHCO_3_ in it. In our research it turned out that *Cl. bifermentans* is able to lactic acid production from glycerol in a wide range of pH values. The results from this experiment are presented in Table 2. Surprisingly, any arranged pH value was lethal to cells of *Cl. bifermentans*. Moreover, even in extremely low and high pH the ability to synthetize a small amount of certain metabolites was maintained. In case of all the investigated strains, in pH 3.0, 10.0 and 13.0, production of 1,3-PD was not observed, while the remaining fermentation products were on a very low level. Unexpectedly, in the above described conditions a relatively large amount of glycerol was used (more than 20%). Probably, in these stressful conditions the cells utilized glycerol as a source of carbon and energy that is needed to prepare them for a sporulation process. The rate of consumption of glycerol and increasing presence of spores during fermentation in pH 3.0 is presented in Table 3. The situations was similar in pH 10.00 and 13.00 The observed optimal pH value to lactic acid production for all the investigated strains of *Cl. bifermentans* was on the lower level than the pH in control fermentation. In case of KM 371, in the pH value 7, the quantity of 19.63 g/L of lactic acid was obtained (Y_LA_=0.39), in the case of KM 374 the pH 8 was an optimal value - 13.88 g/L of lactic acid was obtained (Y_LA_=0.28), while in KM 376 the largest amount of lactic acid was synthetized in pH 6 – 20.92 (Y_LA_=0.42).

**Table 1:** **The metabolite profile of** ***Cl. bifermentans*** **isolates in microerophilic conditions**

Strain	Source of isolation	1,3-PD [g/l]	LA [g/l]	FA	AA [g/l]	SA [g/l]	E [g/l]
*KM 371*	silage	9.71	8.19	1.84	3.81	6.76	1.79
*KM 374*	silage	10.15	8.59	2.28	3.65	0.19	1.43
*KM 376*	silage	7.14	7.52	1.38	2.50	0.50	1.25

1,3-PD, 1,3-propanediol; LA, lactic acid; FA, formic acid; AA, acetic acid; SA, succinic acid; E, ethanol.

**Table 2:** **The metabolite profile of** ***Cl. bifermentans*** **isolates depending on the pH value**

pH/metabolites (g/L)	KM 371							KM 374							KM 376						
	G [%]	1,3-PD [g/L]	SA [g/L]	LA [g/L]	FA [g/L]	AA [g/L]	E [g/L]	G [%]	1,3-PD [g/L]	SA [g/L]	LA [g/L]	FA [g/L]	AA [g/L]	E [g/L]	G [%]	1,3-PD [g/L]	SA [g/L]	LA [g/L]	FA [g/L]	AA [g/L]	E [g/L]
**pH 3.0**	38.22	0.00	0.31	0.55	0.25	0.45	0.00	58.24	0.00	0.63	0.97	0.18	0.85	0.00	43.80	0.00	0.61	0.89	0.17	0.82	0.00
**pH 5.0**	80.22	3.20	1.00	9.89	0.38	1.62	1.79	78.20	4.43	0.91	10.34	0.29	1.30	1.87	72.64	4.43	1.00	10.22	0.25	1.66	1.41
**pH 6.0**	82.00	7.12	1.92	9.93	3.33	4.00	1.61	67.74	6.22	1.21	8.91	0.80	1.77	1.77	97.34	7.91	2.13	20.92	1.36	3.00	0.00
**pH 7.0**	99.82	7.21	1.60	19.63	1.10	2.66	1.71	64.46	8.90	1.00	10.21	1.00	2.01	1.21	69.12	7.79	1.49	16.65	2.26	2.72	0.35
**pH 8.0**	84.62	6.35	0.39	10.96	1.12	1.70	1.75	94.68	6.99	1.34	13.88	1.91	1.65	1.88	85.74	10.18	1.73	10.33	1.94	3.60	0.81
**pH 8.66**	92.45	9.71	6.76	8.19	1.84	3.81	1.79	99.42	10.15	0.19	8.59	2.28	3.65	1.43	95.98	7.14	0.50	7.52	1.38	2.50	1.25
**pH 9.0**	60.24	5.51	0.41	1.05	1.91	1.50	0.36	71.30	6.56	0.40	1.00	0.25	3.50	1.40	71.48	7.34	1.40	1.05	0.31	0.63	0.33
**pH 10.0**	24.68	0.00	0.46	0.79	0.23	0.59	0.00	47.96	0.00	0.38	0.61	0.19	0.50	0.00	43.28	0.00	0.41	0.51	0.31	0.49	0.00
**pH 13.0**	57.82	0.00	0.40	0.57	0.27	0.58	0.00	57.82	0.00	0.40	0.66	0.21	0.61	0.00	68.22	0.00	0.31	0.60	0.21	0.60	0.00

G, the amount of used glycerol; 1,3-PD, 1,3-propanediol; SA, succinic acid; LA, lactic acid; FA, formic acid; AA, acetic acid; E, ethanol.

gly, glycerol; fru, fructose; sor, sorbitol; glu, glucose; man, mannose; mat, mannitol; mal, maltose; xyl, xylose; raf, raffinose; ara, arabinose.

**Table 3:** **The rate of consumption of glycerol and increasing presence of spores during fermentation in pH 3.0**

Time [h]/glycerol/spore	CB 371		CB 374		CB 376	
	G [%]	S [%]	G [%]	S [%]	G [%]	S [%]
**0**	0.00	0.00	0.00	0.00	0.00	0.00
**24**	18.23	21.76	10.87	16.23	8.97	18,75
**48**	28.56	47.24	21.76	47.66	17.87	36,78
**72**	32.55	79.00	24.88	62.02	20.01	49,79
**96**	38.23	100.00	29.12	88.43	21.90	65,35
**120**	38.23	100.00	29.12	100.00	21.90	82,61
**144**	38.23	100.00	29.12	100.00	21.90	100
**170**	38.23	100.00	29.12	100.00	21.90	100

### Influence of the carbon source on the level of lactic acid production

In the next step of the work, the ability of lactic acid production from other carbon sources such as fructose, sorbitol, glucose, mannose, mannitol, maltose, xylose, raffinose, and arabinose was tested. Table 4 shows how the level of lactic acid and other metabolites production changed in this process. The lactic acid was obtained in all fermentations, irrespective of added carbon source to production PY medium (pH 8.66), in the case of all the investigated *Cl. bifermentans* strains. The best results were obtained when mannitol was used: KM 371 synthetized 30.91 g/L (Y_LA_=0.62), 374 synthetized 39.12 g/L (Y_LA_=0.78), and 376 synthetized 38.18 g/L (Y_LA_=0.76) of lactic acid. Good efficiency of lactic acid production was also observed in fermentation of mannose by both KM 374 (Y_LA_=0.59) and KM 376 (Y_LA_=0.63), as well as in fermentation of glucose by KM 376 (Y_LA_=0.57). Surprisingly, during fermentation of no saccharide 1,3-PD was obtained.

**Table 4:** **Effect of various carbon sources on lactic acid and other metabolites production by Cl. bifermentans strains**

Strain/carbon source	KM 371							KM 374							KM 376						
	S [%]	1.3-PD [g/L]	SA [g/L]	LA [g/L]	FA [g/L]	AA [g/L]	E [g/L]	S [%]	1.3-PD	SA [g/L]	LA [g/L]	FA [g/L]	AA [g/L]	E [g/L]	S [%]	1.3-PD [g/L]	SA [g/L]	LA [g/L]	FA [g/L]	AA [g/L]	E [g/L]
**gly**	92.45	9.71	6.76	8.19	1.84	3.81	1.79	99.42	10.15	0.19	8.59	2.28	3.65	1.43	95.98	7.14	0.50	7.52	1.38	2.50	1.25
**fru**	100.00	nd	2.91	16.81	nd	5.35	4.08	78.92	nd	2.40	16.38	1.04	4.89	1.38	65.74	nd	2.27	20.44	nd	5.35	3.32
**sor**	63.16	nd	0.47	21.18	4.14	1.98	4.46	31.38	nd	0.73	5.30	2.53	3.44	7.64	44.56	nd	0.42	21.89	7.34	2.68	4.42
**glu**	100.00	nd	3.17	22.59	nd	5.58	4.31	100.00	nd	2.54	18.47	1.18	5.05	1.48	100.00	nd	3.62	28.52	nd	7.95	3.48
**mann**	100.00	nd	3.11	24.51	nd	6.41	4.13	96.78	nd	3.35	29.09	nd	6.58	4.67	96.48	nd	3.52	31.28	nd	6.57	3.73
**mat**	100.00	nd	1.84	30.91	0.46	1.94	12.54	100.00	nd	3.17	39.12	nd	1.89	3.95	100.00	nd	2.19	38.18	nd	1.89	3.95
**mal**	88.78	nd	1.09	22.65	2.88	2.16	0.87	87.04	nd	1.18	24.85	3.86	2.76	3.46	83.96	nd	nd	23.67	1.34	2.15	0.94
**xyl**	90.84	nd	4.55	7.68	2.14	4.87	2.03	91.38	nd	4.69	4.93	1.95	4.88	1.37	53.74	nd	3.63	4.58	1.48	5.00	1.59
**raf**	100.00	nd	0.87	2.14	0.84	3.00	2.39	16.04	nd	0.21	1.32	0.81	2.37	2.10	45.78	nd	0.72	1.06	1.14	2.23	1.80
**ara**	97.60	nd	4.41	11.00	1.35	6.66	1.90	91.28	nd	4.54	8.33	1.12	5.46	1.08	61.82	nd	2.70	8.13	0.93	4.06	1.27

nd-not detected.

S, the amount of used saccharide; 1.3-PD, 1,3-propanediol; SA, succinic acid; LA, lactic acid; FA, formic acid; AA, acetic acid; E, ethanol.

Gly, glycerol; fru, fructose; sor, sorbitol; glu, glucose; man, mannose; mat, mannitol; mal, maltose; xyl, xylose; raf, raffinose; ara, arabinose.

### Influence of the mixed carbon source on the level of lactic acid production

Because of the ability of KM 371, KM 374 and KM 376 to utilize saccharides such as fructose, sorbitol, glucose, mannose, mannitol, maltose, xylose, raffinose, and arabinose to lactic acid, and better effectiveness of lactic acid synthesis from some of them than from glycerol, the authors decided to investigate the metabolite profile of bacteria when mixed carbon source: glycerol (80% of carbon source) plus other saccharide (20% carbon source) was used. Table 5 shows the results obtained in this experiment. *Cl. bifermentans* strains were able to produce lactic acid in all the media, irrespective of saccharide used as an additional carbon source. Additionally, KM 371, KM 374 and KM 376 were able to 1,3-PD production in almost all the media, except PY supplemented with glycerol plus raffinose as a carbon source in the case of KM 374. The amount of synthetized 1,3-PD was significantly lower than in a medium consisting of glycerol only. Nonetheless, the addition of particular saccharide did not block the metabolic pathway of glycerol to 1,3-PD metabolism. However, mixed carbon sources did not improve previous results of lactic acid obtained by using only mannitol as a carbon source, which gave the best efficiency in its production. Of all the saccharides used, the best results were obtained when medium with glycerol was supplemented with mannitol in the case of KM 371 and CD 376, and with mannose in the case of KM 376. In a medium with glycerol plus mannitol KM 371 synthetized 14.88 g/L (Y_LA_=0.30), and KM 374 produced 14.21 g/L (Y_LA_=0.28) of lactic acid. KM 376 obtained the best efficiency of lactic acid synthesis in a medium with glycerol plus mannose - 16.22 g/L (Y_LA_=0.32). In fact, these results were at a lower level than in a medium consisting of mannitol only, but were similar to a medium with glycerol only. In a medium with glycerol and saccharide, saccharide was preferably used by bacteria cells. The tendency to limit the use of glycerol (in comparison to fermentation with only glycerol as a carbon source) and an increased use of saccharide was observed in all the mixed fermentations. And in effect the saccharides were completely utilized.

**Table 5:** **Effect of mixed carbon sources on lactic acid and other metabolites production by** ***Cl. bifermentans*** **strains**

Strain/carbon source	KM 371							KM 374							KM 376						
	G/S [%]	1.3-PD [g/L]	SA [g/L]	LA [g/L]	FA [g/L]	AA [g/L]	E [g/L]	G/S [%]	G/S [%]	1.3-PD [g/L]	SA [g/L]	LA [g/L]	FA [g/L]	AA [g/L]	G/S [%]	1.3-PD [g/L]	SA [g/L]	LA [g/L]	FA [g/L]	AA [g/L]	E [g/L]
**gly -**	92.45 -	9.71	6.76	8.19	1.84	3.81	1.79	99.42/-	10.15	0.19	8.59	2.28	3.65	1.43	95.98/-	7.14	0.50	7.52	1.38	2.50	1.25
**gly + fru**	46.48/99.98	0.95	0.92	9.18	0.54	1.08	1.33	48.86/80.66	1.16	nd	7.59	0.57	1.06	0.72	57.38/99.98	3.27	1.01	9.69	0.00	1.61	0.99
**gly + sor**	67.65/78.96	4.92	nd	7.16	0.81	1.81	1.17	40.98/77.34	nd	nd	2.98	1.73	0.32	2.52	72.25/99.99	8.32	0.66	9.56	1.25	2.61	0.74
**gly + glu**	53.08/99.98	1.54	0.89	10.29	nd	1.16	1.34	41.80/99.98	1.03	nd	10.28	0.64	1.34	1.12	54.32/99.98	3.60	0.93	11.82	nd	1.69	1.12
**gly + man**	51.98/90.58	1.76	0.63	10.30	0.57	1.11	1.26	56.35/98.65	1.76	nd	9.59	0.73	1.18	0.95	45.12/99.98	4.42	1.13	16.22	nd	2.13	0.69
**gly + mat**	30.03/99.99	0.98	0.62	14.88	2.28	2.12	0.70	38.80/99.99	1.07	1.48	14.21	1.07	2.06	0.77	69.80/99.99	3.54	1.47	14.43	1.29	1.78	0.68
**gly + mal**	62.18/99.97	2.20	0.64	8.89	1.15	0.88	0.82	46.60/96.96	2.81	0.62	10.95	1.16	1.11	1.01	57.07/99.97	3.58	1.09	10.86	nd	1.52	0.94
**gly + xyl**	62.00/99.84	3.39	0.80	7.94	1.08	1.14	1.33	55.02/99.98	2.30	0.77	7.11	0.90	0.94	1.03	67.35/99.98	7.84	1.30	12.02	1.09	2.48	0.78
**gly + raf**	63.45/100.00	3.99	0.58	7.01	0.62	1.46	1.07	45.40/100.00	nd	0.58	0.87	0.44	0.17	0.51	67.13/100.00	5.83	nd	7.66	0.82	1.82	1.42
**gly + ara**	77.65/99.99	3.28	0.63	6.99	0.76	0.92	1.11	63.80/99.98	3.37	0.69	7.40	0.80	1.04	1.32	81.15/99.99	9.85	1.48	12.19	0.87	2.80	0.67

nd-not detected.

G, the amount of used glycerol; S, the amount of used saccharide; 1,3-PD, 1,3-propanediol; SA, succinic acid; LA, lactic acid; FA, formic acid; AA, acetic acid; E, ethanol.

Gly, glycerol; fru, fructose; sor, sorbitol; glu, glucose; man, mannose; mat, mannitol; mal, maltose; xyl, xylose; raf, raffinose; ara, arabinose.

## Discussion

The natural environment is a good source of industrially useful strains. In our previous work we isolated from the environmental probe the new bacteria strains able to 1,3-PD from glycerol (Leja et al. 2011), which have a variety of industrial applications, such as chemical intermediates used in the manufacture of polymers, cosmetics, medicines and heterocyclic compounds (Kośmider et al. 2011). During that experiment we obtained *Cl. bifermentans* strains which were not known as 1,3-PD producers yet. It occurred that these isolates are also able to produce another industrially useful metabolite from glycerol, lactic acid (Myszka et al. 2012), which is widely used in the food, cosmetic, pharmaceutical, and chemical industries and has received increased attention for potential use as a monomer in the production of biodegradable poly (lactic acid) (Wee et al. 2006). In the existing literature there is no information that the species of *Cl. bifermentans* is able to lactic acid synthesis. Generally, the metabolite profile of the species of *Cl. bifermentans* is not investigated sufficiently as yet. Thus the present authors decided to investigate into lactic acid production by these species. Our work’s aim was to check whether or not a medium pH and a carbon source exert an influence on lactic acid production by *Cl. bifermentans* strains. Some scientists argued that *Cl. bifermentans* exhibit adaptability in extreme environmental conditions (Lauro et al. 2004) and that they are able to survive in extreme pH levels (Sengupta et al. 2011). Moreover, Gibbs (1964) stated that even incubation at pH 10.0 or pH 3.0 has no significant effects on the ability of spores of *Cl. bifermentans* to germinate and that the vegetative cells are able to survive in these extreme conditions. We decided thus to investigate changes in metabolite profiles depending on the pH level of a fermentative medium which includes radical values such as 3 or 13. It occurred that the decreasing of pH value to 8.0, 7.0, and 6.0 results in the increased yield of lactic acid production. The data presented in the existing literature confirms this observation: lactic acid production requires strict control of the pH, mostly at values between 6 and 8 (Kascak et al. 1996; Litchfield 1996; Hofvendahl & Hahn-Hägerdal 2000). For example, the optimal pH for lactic acid synthesis for *Lactobacillus bulgaricus* is 6.0 (Venkatesh et al. 1993) and for *Lactobacillus casei* is 6.5 (Panesar et al. 2010). Our isolates prefer pH 7 (KM 371 and KM 374) and 6 (KM 376).

We selected glycerol for our research on microbiological production of industrially useful metabolites because a significant increase in biodiesel production was observed within the last decade (Kośmider et al. 2011). Presently, the most often used biodiesel fuels are vegetable oil fatty acid methyl or ethyl esters produced by transesterification. For every three molls of ethyl esters one mol of crude glycerol is produced, which is an equivalent to approximately 10% of total biodiesel production (Kośmider et al. 2011; Rahman et al. 2002). It is estimated that by 2016 the world biodiesel market will achieve the quantity of 37 billion gallons, which means that much more than 4 billion gallons of crude glycerol will be produced every year (Kośmider et al. 2011). Accordingly, it is necessary to find a new effective method to utilized this amount of crude glycerol. The research on production 1,3-PD from crude glycerol by microbiological way is extensively described worldwide e.g., (Hiremath et al. 2011; Mendes et al. 2011; Vaidyanathan et al. 2011; Chatzifragkou et al. 2011; Wilkens et al. 2012; Ringel et al. 2012), but only a few papers concern the production of lactic acid from this by-product, and moreover, publications concentrate mainly on genetic engineered strains (Posada et al. 2012; Ruhal & Choudhury 2012); at the same time only a few papers discuss lactic acid production from other renewable resources (Hofvendahl & Hahn-Hägerdal 2000; Yadav et al. 2011). During our work it occurred that *Cl. bifermentans* strains are indeed able to synthesize lactic acid from glycerol. The yields of lactic acid for KM 371, KM 374, and KM 376 were, respectively, Y_LA_=0.16, Y_LA_=0.17, and Y_LA_=0.17. These values are lower than the ones quoted in the work by (Ruhal & Choudhury 2012) on the mutant of *Propionibacterium freudenreichii* subspp. *shermanii* in which they obtained Y_LA_=0.3. However, in our work the bacteria utilized more glycerol (68.22%, 79.08%, and 80.22%, respectively) than in the above mentioned work, in which only 25.00% was consumed. Our results in the yield of lactic acid obtained by isolates of *Cl. bifermentans* were comparable with the results obtained in other investigations in which some kinds of variable renewable resources were used, such as a carbon source; e.g., in the case of lactic acid from whey permeate by *Lactobacillus lactis* sp. *lactis* 2432 Y_LA_=0.21, from solid waste by *Lb. lactis* sp. *lactis* NRRL B-4449 Y_LA_=0.16, and from wheat flour hydrolyzed by *Lb. delbrueckii* sp. *bulgaricus* ATCC 11842 Y_LA_=0.11 (Hofvendahl & Hahn-Hägerdal 2000).

In the literature there is a lot of information about lactic acid production from other carbon sources such as saccharides e.g., (Wee et al. 2006; Hujanen et al. 2001; Liu 2003; Jun et al. 2003). Thus we wanted to check if the change of carbon source from glycerol to pure saccharides increases the level of lactic acid synthesis by *Cl. bifermentans* isolates. It occurred that the highest productions of lactic acid were obtained when mannitol was used – the yield of production increased more than three times: Y_LA_=0.62, Y_LA_=0.78, and Y_LA_=0.76 in the case of KM 371, KM 374, and KM 376, respectively. Moreover, some lactic acid bacteria, as it turned out, are able to ferment mannitol into lactic acid. For instance, *Lactobacillus casei* utilizes mannitol through the following pathway: mannitol->mannitol-1-phosphate->fructose-6-phosphate->2 pyruvate->2 lactate (Liu 2003). Under aerobic conditions, *Lb. casei* converts mannitol primarily to lactate only. However, under anaerobic conditions mannitol is fermented to lactate, acetate, formate, and ethanol (Liu 2003). The effect of variable saccharides on the lactic acid production by *Rhizopus oryzae* was investigated in the work by Yin et al. (1997). These authors tested the efficiency of lactic acid production from glucose, mannose, fructose, sucrose, raffinose, inulin, maltose, rhamnose, xylose, galactose, and corn starch. It occurred that mannitol is a good carbon source also in lactic acid production by *Rhizopus oryzae* and the Y_LA_=0.70 which is comparable with the results obtained in the present work. This step of our experiment also shows that all the saccharides used (except of xylose, raffinose and, additionally, sorbitol in the case of KM 374) are a preferable carbon source for lactic acid synthesis. The main aim of this work, however, was to investigate into how utilize glycerol as a by-product from biodiesel production. Thus it was checked if the addition of small amount of saccharide to glycerol used as a main carbon source can result in an increase of the level of lactic acid synthetized. Generally, the levels of lactic acid obtained from a mixed carbon source were comparable with the results from our tests with glycerol only. Only in the case of addition of mannitol for all strains, and mannose for KM 376, the yields of lactic acid increased. When some saccharides were used as a carbon source – no 1,3-PD was synthetized, in the situation when the saccharides were added to glycerol, 1,3-PD was synthetized. Moreover, the amount of utilized glycerol was lower and the saccharides were completely consumed. Biebl and Marten (1995) made similar observations. In their experiments, glucose was applied in half the concentration of glycerol for a mixed-substrate culture. It occurred that the addition of glycerol to medium with glucose increased the rate of glucose utilization by *Cl. butyricum* (up to 8 h). Moreover, product formation changed markedly in comparison with glycerol fermentation as 90% of the glycerol was converted to 1,3-PD and only 10% was used for acids. Additionally, mixed fermentation (glycerol plus glucose) shifted from butyrate to acetate production. Because the addition of the saccharides did not increase the efficiency of lactic acid production, a better solution from the environmental point of view is to optimize the production of metabolites using only glycerol as a carbon source. Growing prices of crude oil and fuels for the transportation sectors have resulted in a rapid growth in biodiesel production worldwide. An increase of biodiesel production leads thus to an increased quantity of its primary co-product, glycerol. Since the existing glycerol supply and demand market was tight, the recent increase in glycerol production from biodiesel refining has created a glut in the glycerol market. This situation made the price of glycerol fall significantly and biodiesel refiners are faced with limited options for managing the glycerol by-product (Johnson & Taconi 2007). One of the solutions of this problem is to use crude glycerol in the production of industrially useful metabolites such as lactic acid and 1,3-PD (Kubiak et al. 2012).

## Authors’ original submitted files for images

Below are the links to the authors’ original submitted files for images. Authors’ original file for figure 1 Authors’ original file for figure 2 Authors’ original file for figure 3 Authors’ original file for figure 4 Authors’ original file for figure 5

## References

[CR1] Bergey DH, Harrison FC, Breed RS, Hammer BW, Huntoon FM (1923). Bergey’s Manual of Determinative Bacteriology.

[CR2] Biebl H, Marten S (1995). Fermentation of glycerol to 1,3-propanediol: use of co substrates. Appl Microbiol Biotechnol.

[CR3] Biebl H, Spröer C (2002). Taxonomy of the glycerol fermenting Clostridia and description of Clostridium diolis sp. nov. syst. Appl Microbiol.

[CR4] Brooks E, Epps HBG (1958). Taxonomic studies of the genus Clostridium: Clostridium bifermentans and Clostridium sordelli. J Gen Microbiol.

[CR5] Chatzifragkou A, Papanikolaou S, Dietz D, Doulgeraki AI, Nychas GJE, Zeng AP (2011). Production of 1,3-propanediol by Clostridium butyricum growing on biodiesel-derived crude glycerol through a non-sterilized fermentation process. Appl Microbiol Biotechnol.

[CR6] Clark FE, Hall IC (1937). A comparative study of Bacillus bifermentans (Tissier and Martelly), Bacillus centrosporogenes (Hall) and certain closely related proteolytic anaerobes. J Bacteriol.

[CR7] Gibbs PA (1964). Factors affecting the germination of spores of Clostridium bifermentans. J Gen Microbial.

[CR8] Hiremath A, Kannabiran M, Rangaswamy V (2011). 1,3-Propanediol production from crude glycerol from jatropha biodiesel process. New Biotechnol.

[CR9] Hofvendahl K, Hahn-Hägerdal B (2000). Factors affecting the fermentative lactic acid production from renewable resources. Enzyme Microbial Technol.

[CR10] Hujanen M, Linko S, Linko YY, Leisola M (2001). Optimization of media and cultivation conditions for L(+)(S)-lactic acid production by Lactobacillus casei NRRL B-441. Appl Mirobiol Biotechnol.

[CR11] Johnson DT, Taconi KA (2007). The glycerin glut: options for the value-added conversion of crude glycerol resulting from biodiesel production. Environ Prog.

[CR12] Jun JS, Wee YJ, Ryu HW (2003). Production of optically pure l(+)-lactic acid from various carbohydrates by batch fermentation of Enterococcus faecalis RKY1. Enzyme Microb Technol.

[CR13] Kascak JS, Kominek J, Roehr M, Rehm HJ, Reed G, Stadler AP (1996). Lactic acid. Biotechnology Biotransformations.

[CR14] Khanal SM, Chen WH, Sung S (2004). Biological hydrogen production: effects of pH and intermediate products. Int J Hydrogen Energy.

[CR15] Kośmider A, Leja K, Czaczyk K, Montero G, Stoytchera M (2011). Improved utilization of crude glycerol by-product from biodiesel production. Biodiesel – Quality, Emissions and By-Products.

[CR16] Kubiak P, Leja K, Myszka K, Celińska E, Spychała M, Szymanowska-Powałowska D, Czaczyk K, Grajek W (2012). Physiological predisposition of various Clostridium species to synthetize 1,3-propanediol. Process Biochem.

[CR17] Lauro FM, Bertoloni G, Obraztsova A, Kato C, Tebo BM, Bartlett DH (2004). Pressure effects on Clostridium strains isolated from a cold deep-sea environment. Extremophiles.

[CR18] Leja K, Myszka K, Kubiak P, Wojciechowska J, Olejnik-Schmidt AK, Czaczyk K, Grajek W (2011). Isolation and identification of Clostridium spp. from natural samples that performs effective conversion of glycerol to 1,3-propanediol. Acta Scientarium Polonorum - Biotechnologia.

[CR19] Levin BD, Islam R, Cicek N, Sparling R (2006). Hydrogen production by Clostridium thermocellum 27405 from cellulosic biomass substrates. Int J Hydrogen Energy.

[CR20] Litchfield JH (1996). Microbiological production of lactic-acid. Adv Appl Microbiol.

[CR21] Liu SQ (2003). Practical implications of lactate and pyruvate metabolism by lactic acid bacteria in food and beverage fermentations. Int J Food Microb.

[CR22] Mendes FS, González-Pajuelo M, Cordier H, François JM, Vasconcelos I (2011). 1,3-Propanediol production in a two-step process fermentation from renewable feedstock. Appl Microb Biotech.

[CR23] Myszka K, Leja K, Olejnik-Schmidt A, Czaczyk K (2012). Isolation process of industrially useful Clostridium bifermentans from natural samples. J Biosc Bioengin.

[CR24] Nachman S, Kaul A, Li KI, Slim MS, Filippo JA, Horn KV (1989). Liver abscess caused by Clostridium bifermentans following blunt abdominal trauma. J Clin Microbiol.

[CR25] Panesar PS, Kennedy JF, Knill CJ, Kosseva M (2010). Production of L(+) lactic acid using Lactobacillus casei from whey. Braz arch biol technol.

[CR26] Posada JA, Cardona CA, Gonzalez R (2012). Analysis of the production process of optically pure d-lactic acid from raw glycerol using engineered Escherichia coli strains. Appl Microbiol Biotechnol.

[CR27] Rahman KSM, Rahman TJ, McClean S, Marchant R, Bant IM (2002). Rhamnolipid biosurfactant production by strains of Pseudomonas aeruginosa using low-cost raw materials. Biotechnol Progress.

[CR28] Ringel AK, Wilkens E, Hortig D, Willke T, Vorlo KD (2012). An improved screening method for microorganisms able to convert crude glycerol to 1,3-propanediol and to tolerate high product concentrations. Appl Microbiol Biotechnol.

[CR29] Ruhal R, Choudhury B (2012). Use of an osmotically sensitive mutant of Propionibacterium freudenreichii subspp. shermanii for the simultaneous productions of organic acids and trehalose from biodiesel waste based crude glycerol. Bior Technol.

[CR30] Sengupta N, Alama SI, Kumara RB, Singh L (2011). Diversity and antibiotic susceptibility pattern of cultivable anaerobic bacteria from soil and sewage samples of India. Inf Gen Evol.

[CR31] Vaidyanathan H, Kandasamy V, Ramakrishnan GG, Ramachandran KB, Jayaraman G, Ramalingam S (2011). Glycerol conversion to 1,3-Propanediol is enhanced by the expression of a heterologous alcohol dehydrogenase gene in Lactobacillus reuteri. Chem Mat Science.

[CR32] Venkatesh KV, Okos MR, Wankat PC (1993). Kinetic model of growth and lactic acid production from lactose by Lactobacillus bulgaricus. Process Biochem.

[CR33] Wang CC, Chang CW, Chu CP, Lee DJ, Chang BV, Liao CS (2003). Producing hydrogen from wastewater sludge by Clostridium bifermentans. J Biotechnol.

[CR34] Wee YJ, Kim JN, Ryu HW (2006). Biotechnological production of lactic acid. Food Technol Biotechnol.

[CR35] Wilkens E, Ringel AK, Hortig D, Willke T, Vorlop KD (2012). High-level production of 1,3-propanediol from crude glycerol by Clostridium butyricum AKR102a. Appl Microbiol Biotechnol.

[CR36] Wu Z, Yang SH (2003). Extractive fermentation for butyric acid production from glucose by Clostridium tyrobutyricum. Biot Bioengin.

[CR37] Yadav AK, Chaudhari A, Kothari RM (2011). Bioconversion of renewable resources into lactic acid: an industrial view. Critical Rev Biotechnol.

[CR38] Yin P, Nishina N, Kosakai Y, Yahiro K, Park Y, Okabe M (1997). Enhanced production of L(+)-lactic acid from corn starch in a culture of Rhizopus oryzae using an air-lift bioreactor. J Ferment Bioengin.

