# Supplementary material for: The ability of Clostridium bifermentans strains to lactic acid biosynthesis in various environmental conditions
Source: Springerplus. 2013 Feb 11;2(1):44. doi: 10.1186/2193-1801-2-44 (PMC3595471; doi:10.1186/2193-1801-2-44)
Supplement: Supplementary file 1 — Authors’ original file for figure 1 [file 40064_2012_118_MOESM1_ESM.pdf]

Table 2 The metabolite profile of *Cl. bifermentans* isolates depending on the pH value

	KM 371							KM 374							KM 376						
**pH/ metabolites (g/L)**	G [%]	1,3-PD [g/L]	SA [g/L]	LA [g/L]	FA [g/L]	AA [g/L]	E [g/L]	G [%]	1,3-PD [g/L]	SA [g/L]	LA [g/L]	FA [g/L]	AA [g/L]	E [g/L]	G [%]	1,3-PD [g/L]	SA [g/L]	LA [g/L]	FA [g/L]	AA [g/L]	E [g/L]
**pH 3.0**	38.22	0.00	0.31	0.55	0.25	0.45	0.00	58.24	0.00	0.63	0.97	0.18	0.85	0.00	43.80	0.00	0.61	0.89	0.17	0.82	0.00
**pH 5.0**	80.22	3.20	1.00	9.89	0.38	1.62	1.79	78.20	4.43	0.91	10.34	0.29	1.30	1.87	72.64	4.43	1.00	10.22	0.25	1.66	1.41
**pH 6.0**	82.00	7.12	1.92	9.93	3.33	4.00	1.61	67.74	6.22	1.21	8.91	0.80	1.77	1.77	97.34	7.91	2.13	20.92	1.36	3.00	0.00
**pH 7.0**	99.82	7.21	1.60	19.63	1.10	2.66	1.71	64.46	8.90	1.00	10.21	1.00	2.01	1.21	69.12	7.79	1.49	16.65	2.26	2.72	0.35
**pH 8.0**	84.62	6.35	0.39	10.96	1.12	1.70	1.75	94.68	6.99	1.34	13.88	1.91	1.65	1.88	85.74	10.18	1.73	10.33	1.94	3.60	0.81
**pH 8.66**	92.45	9.71	6.76	8.19	1.84	3.81	1.79	99.42	10.15	0.19	8.59	2.28	3.65	1.43	95.98	7.14	0.50	7.52	1.38	2.50	1.25
**pH 9.0**	60.24	5.51	0.41	1.05	1.91	1.50	0.36	71.30	6.56	0.40	1.00	0.25	3.50	1.40	71.48	7.34	1.40	1.05	0.31	0.63	0.33
**pH 10.0**	24.68	0.00	0.46	0.79	0.23	0.59	0.00	47.96	0.00	0.38	0.61	0.19	0.50	0.00	43.28	0.00	0.41	0.51	0.31	0.49	0.00
**pH 13.0**	57.82	0.00	0.40	0.57	0.27	0.58	0.00	57.82	0.00	0.40	0.66	0.21	0.61	0.00	68.22	0.00	0.31	0.60	0.21	0.60	0.00

G – the amount of used glycerol; 1,3-PD – 1,3-propanediol; SA – succinic acid; LA – lactic acid ; FA –formic acid ; AA acetic acid; E – ethanol

gly – glycerol; fru – fructose; sor – sorbitol; glu – glucose; man – mannose; mat – mannitol; mal – maltose; xyl – xylose; raf – raffinose; ara - arabinose
