# Supplementary material for: The ability of Clostridium bifermentans strains to lactic acid biosynthesis in various environmental conditions
Source: Springerplus. 2013 Feb 11;2(1):44. doi: 10.1186/2193-1801-2-44 (PMC3595471; doi:10.1186/2193-1801-2-44)
Supplement: Supplementary file 2 — Authors’ original file for figure 2 [file 40064_2012_118_MOESM2_ESM.pdf]

Table 4 Effect of various carbon sources on lactic acid and other metabolites production by *Cl. bifermentans* strains

strain/ carbon source	KM 371							KM 374							KM 376						
	S [%]	1.3-PD [g/L]	SA [g/L]	LA [g/L]	FA [g/L]	AA [g/L]	E [g/L]	S [%]	1.3-PD	SA [g/L]	LA [g/L]	FA [g/L]	AA [g/L]	E [g/L]	S [%]	1.3-PD [g/L]	SA [g/L]	LA [g/L]	FA [g/L]	AA [g/L]	E [g/L]
**gly**	92.45	9.71	6.76	8.19	1.84	3.81	1.79	99.42	10.15	0.19	8.59	2.28	3.65	1.43	95.98	7.14	0.50	7.52	1.38	2.50	1.25
**fru**	100.00	nd	2.91	16.81	nd	5.35	4.08	78.92	nd	2.40	16.38	1.04	4.89	1.38	65.74	nd	2.27	20.44	nd	5.35	3.32
**sor**	63.16	nd	0.47	21.18	4.14	1.98	4.46	31.38	nd	0.73	5.30	2.53	3.44	7.64	44.56	nd	0.42	21.89	7.34	2.68	4.42
**glu**	100.00	nd	3.17	22.59	nd	5.58	4.31	100.00	nd	2.54	18.47	1.18	5.05	1.48	100.00	nd	3.62	28.52	nd	7.95	3.48
**mann**	100.00	nd	3.11	24.51	nd	6.41	4.13	96.78	nd	3.35	29.09	nd	6.58	4.67	96.48	nd	3.52	31.28	nd	6.57	3.73
**mat**	100.00	nd	1.84	30.91	0.46	1.94	12.54	100.00	nd	3.17	39.12	nd	1.89	3.95	100.00	nd	2.19	38.18	nd	1.89	3.95
**mal**	88.78	nd	1.09	22.65	2.88	2.16	0.87	87.04	nd	1.18	24.85	3.86	2.76	3.46	83.96	nd	nd	23.67	1.34	2.15	0.94
**xyl**	90.84	nd	4.55	7.68	2.14	4.87	2.03	91.38	nd	4.69	4.93	1.95	4.88	1.37	53.74	nd	3.63	4.58	1.48	5.00	1.59
**raf**	100.00	nd	0.87	2.14	0.84	3.00	2.39	16.04	nd	0.21	1.32	0.81	2.37	2.10	45.78	nd	0.72	1.06	1.14	2.23	1.80
**ara**	97.60	nd	4.41	11.00	1.35	6.66	1.90	91.28	nd	4.54	8.33	1.12	5.46	1.08	61.82	nd	2.70	8.13	0.93	4.06	1.27

nd-not detected

S - the amount of used saccharide; 1.3-PD – 1,3-propanediol; SA – succinic acid; LA – lactic acid; FA –formic acid; AA acetic acid; E – ethanol
gly – glycerol; fru – fructose; sor – sorbitol; glu – glucose; man – mannose; mat – mannitol; mal – maltose; xyl – xylose; raf – raffinose; ara - arabinose
