# Supplementary material for: The ability of Clostridium bifermentans strains to lactic acid biosynthesis in various environmental conditions
Source: Springerplus. 2013 Feb 11;2(1):44. doi: 10.1186/2193-1801-2-44 (PMC3595471; doi:10.1186/2193-1801-2-44)
Supplement: Supplementary file 3 — Authors’ original file for figure 3 [file 40064_2012_118_MOESM3_ESM.pdf]

**Table 5** Effect of mixed carbon sources on lactic acid and other metabolites production by *Cl. bifermentans* strains

| strain/ carbon source | KM 371 | | | | | | | KM 374 | | | | | | | KM 376 | | | | | | |
|---|---|---|---|---|---|---|---|---|---|---|---|---|---|---|---|---|---|---|---|---|---|
| | G/S [%] | 1.3-PD [g/L] | SA [g/L] | LA [g/L] | FA [g/L] | AA [g/L] | E [g/L] | G/S [%] | G/S [%] | 1.3-PD [g/L] | SA [g/L] | LA [g/L] | FA [g/L] | AA [g/L] | G/S [%] | 1.3-PD [g/L] | SA [g/L] | LA [g/L] | FA [g/L] | AA [g/L] | E [g/L] |
| **gly -** | 92.45 - | 9.71 | 6.76 | 8.19 | 1.84 | 3.81 | 1.79 | 99.42/ - | 10.15 | 0.19 | 8.59 | 2.28 | 3.65 | 1.43 | 95.98/ - | 7.14 | 0.50 | 7.52 | 1.38 | 2.50 | 1.25 |
| **gly+ fru** | 46.48/ 99.98 | 0.95 | 0.92 | 9.18 | 0.54 | 1.08 | 1.33 | 48.86/ 80.66 | 1.16 | nd | 7.59 | 0.57 | 1.06 | 0.72 | 57.38/ 99.98 | 3.27 | 1.01 | 9.69 | 0.00 | 1.61 | 0.99 |
| **gly+ sor** | 67.65/ 78.96 | 4.92 | nd | 7.16 | 0.81 | 1.81 | 1.17 | 40.98/ 77.34 | nd | nd | 2.98 | 1.73 | 0.32 | 2.52 | 72.25/ 99.99 | 8.32 | 0.66 | 9.56 | 1.25 | 2.61 | 0.74 |
| **gly+ glu** | 53.08/ 99.98 | 1.54 | 0.89 | 10.29 | nd | 1.16 | 1.34 | 41.80/ 99.98 | 1.03 | nd | 10.28 | 0.64 | 1.34 | 1.12 | 54.32/ 99.98 | 3.60 | 0.93 | 11.82 | nd | 1.69 | 1.12 |
| **gly+ man** | 51.98/ 90.58 | 1.76 | 0.63 | 10.30 | 0.57 | 1.11 | 1.26 | 56.35/ 98.65 | 1.76 | nd | 9.59 | 0.73 | 1.18 | 0.95 | 45.12/ 99.98 | 4.42 | 1.13 | 16.22 | nd | 2.13 | 0.69 |
| **gly+ mat** | 30.03/ 99.99 | 0.98 | 0.62 | 14.88 | 2.28 | 2.12 | 0.70 | 38.80/ 99.99 | 1.07 | 1.48 | 14.21 | 1.07 | 2.06 | 0.77 | 69.80/ 99.99 | 3.54 | 1.47 | 14.43 | 1.29 | 1.78 | 0.68 |
| **gly+ mal** | 62.18/ 99.97 | 2.20 | 0.64 | 8.89 | 1.15 | 0.88 | 0.82 | 46.60/ 96.96 | 2.81 | 0.62 | 10.95 | 1.16 | 1.11 | 1.01 | 57.07/ 99.97 | 3.58 | 1.09 | 10.86 | nd | 1.52 | 0.94 |
| **gly+ xyl** | 62.00/ 99.84 | 3.39 | 0.80 | 7.94 | 1.08 | 1.14 | 1.33 | 55.02/ 99.98 | 2.30 | 0.77 | 7.11 | 0.90 | 0.94 | 1.03 | 67.35/ 99.98 | 7.84 | 1.30 | 12.02 | 1.09 | 2.48 | 0.78 |
| **gly+ raf** | 63.45/ 100.00 | 3.99 | 0.58 | 7.01 | 0.62 | 1.46 | 1.07 | 45.40/ 100.00 | nd | 0.58 | 0.87 | 0.44 | 0.17 | 0.51 | 67.13/ 100.00 | 5.83 | nd | 7.66 | 0.82 | 1.82 | 1.42 |
| **gly+ ara** | 77.65/ 99.99 | 3.28 | 0.63 | 6.99 | 0.76 | 0.92 | 1.11 | 63.80/ 99.98 | 3.37 | 0.69 | 7.40 | 0.80 | 1.04 | 1.32 | 81.15/ 99.99 | 9.85 | 1.48 | 12.19 | 0.87 | 2.80 | 0.67 |

nd-not detected

G – the amount of used glycerol; S - the amount of used saccharide; 1,3-PD – 1,3-propanediol; SA – succinic acid; LA – lactic acid; FA –formic acid; AA acetic acid; E – ethanol
gly – glycerol; fru – fructose; sor – sorbitol; glu – glucose; man – mannose; mat – mannitol; mal – maltose; xyl – xylose; raf – raffinose; ara - arabinose
